# Inverse Association between Oxidative Balance Score and Incident Type 2 Diabetes Mellitus

**DOI:** 10.3390/nu15112497

**Published:** 2023-05-27

**Authors:** Yu-Jin Kwon, Hye-Min Park, Jun-Hyuk Lee

**Affiliations:** 1 Department of Family Medicine, Yongin Severance Hospital, Yonsei University College of Medicine, Yongin 16995, Republic of Korea; digda3@yuhs.ac; 2 Primary Care Research Center, National Health Insurance Service, Ilsan Hospital, Goyang 10326, Republic of Korea; jadorehm@nhimc.or.kr; 3 Department of Family Medicine, Nowon Eulji Medical Center, Eulji University School of Medicine, Seoul 01830, Republic of Korea; 4 Department of Medicine, School of Medicine, Hanyang University, Seoul 04763, Republic of Korea

**Keywords:** oxidative balance score, antioxidant, type 2 diabetes mellitus, Korean genome, epidemiology study

## Abstract

Mitigating the risk of type 2 diabetes mellitus (T2DM) can be achieved through the maintenance of a healthy weight, the adoption of a healthy diet, and engaging in regular physical activity. The oxidative balance score (OBS), an integrated measure of pro- and antioxidant exposure conditions, represents an individual’s overall oxidative balance status. This study aimed to evaluate the association between OBS and T2DM incidence using data from a large, community-based, prospective cohort study. Data from 7369 participants aged 40–69 years who engaged in the Korean Genome and Epidemiology Study (KoGES) were analyzed. The hazard ratio (HR) and 95% confidence interval (CI) for T2DM incidence of sex-specific OBS tertile groups were calculated using univariable and multivariable Cox proportional hazard regression analyses. During the mean 13.6-year follow-up period, 908 men and 880 women developed T2DM. The fully-adjusted HR (95% CI) for incident T2DM of the middle and highest tertile groups, compared with the referent lowest tertile group, were 0.86 (0.77–1.02) and 0.83 (0.70–0.99) in men and were 0.94 (0.80–1.11) and 0.78 (0.65–0.94) in women, respectively. Individuals with a high OBS are at lower risk for the development of T2DM. This implies that lifestyle modification with more antioxidant properties could be a preventive strategy for T2DM.

## 1. Introduction

Globally, the number of persons with diabetes mellitus is rising, with the International Diabetes Federation estimating that there were 463 million cases of the disease in 2019 and that there would be 700.2 million cases by 2045 [1]. The prevalence of type 2 diabetes mellitus (T2DM) among South Korean adults rapidly increased over the past decades from 1.5% in 1971 to 13.7% in 2016 [2]. The economic burden of diabetes mellitus in Korea was USD 18,293 million in 2019 [3]. Moreover, the per capita cost increased nearly four times, from USD 3991 to USD 11,965, when the number of complications due to diabetes mellitus increased from one to three or more [3]. Patients with T2DM are at a higher risk of cardiovascular mortality, all-cause mortality, and comorbidities, including cardiovascular disease, cerebrovascular disease, and peripheral vascular disease [4]. Therefore, preventive strategies for T2DM have been emphasized to reduce this disease burden [4].

The two main characteristics of T2DM are target tissue insulin resistance and a relative deficiency of insulin production from pancreatic β-cells [5]. Over recent years, numerous studies have demonstrated a synergistic interaction between inflammation-related insulin resistance [6]. The emerging role of chronic low-grade inflammation in insulin resistance and β-cell dysfunction in T2DM has engendered increasing attention in targeting inflammation to advance the prevention and management of the disease [7].

A recent meta-analysis elucidated that a coalescence of low-risk lifestyle behaviors (such as appropriate body weight, healthy eating habits, light alcohol consumption, regular exercise, and smoking cessation) resulted in an 80% reduction in the risk of developing T2DM [8]. This finding aligns with a previous study emphasizing the balance between antioxidants and oxidative stress in chronic diseases [9]. Smoking is a powerful pro-oxidant, and the burden of oxidative stress could be exacerbated through the secondary release of oxygen radicals from inflammation status [9]. Therefore, several studies proposed a link between chronic disease and the oxidative balance score (OBS) [10,11,12,13,14,15,16,17]. The OBS evaluates the oxidative balance of the lifestyle pattern of a subject in terms of the incorporated consumption of anti- and pro-oxidants [10,12,18]. Lifestyle (cigarette smoking and alcohol drinking), healthy body weight (obesity and abdominal obesity), and healthy diet (lower intakes of saturated fatty acid [SFA], omega-6 poly-unsaturated fatty acid [PUFA], iron and high intakes of vitamin C, vitamin E, omega-3 PUFA, selenium, and beta-carotene) could be involved as OBS components [10].

To the best of our knowledge, no previous study has comprehensively examined OBS and the incidence of T2DM in the middle-aged and elderly. Therefore, we prospectively investigated the development of T2DM according to the OBSs of tertile groups from a large-population, community-based Korean cohort observed over 16 years.

## 2. Materials and Methods

### 2.1. Study Population

**Figure 1:**
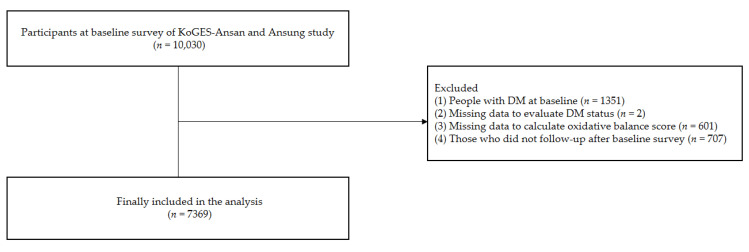
Flowchart of the study population selection.

We used the Korean Genome and Epidemiology Study (KoGES)-Ansan and Ansung, embedded in the KoGES, a large, community-based study in Korea. The study design and procedures were detailed in a previous study [19]. KoGES-Ansan and Ansung included 10,030 adults aged from 40 to 69 years. This survey was conducted between 2001 and 2002 and followed up every two years. For the present study, we included participants with the eighth follow-up, conducted between 2017 and 2018. Initially, a total of 10,030 participants received a health examination and questionnaire. Subsequently, we excluded participants with T2DM at baseline (*n* = 1351), those with missing data to evaluate T2DM (*n* = 2), those with missing data to calculate OBSs (*n* = 601), and those who did not follow up after the baseline survey (*n* = 707). Finally, a total of 7369 participants (3485 men and 3884 women) were included in this analysis. The flow chart is shown in Figure 1. All participants in this study provided informed consent. This study protocol was approved by the Nowon Eulji Medical Center’s Institutional Review Board (approval number: 2021-09-025) and followed the ethical criteria of the 1964 Declaration of Helsinki and its subsequent amendments.

### 2.2. Assessment of Oxidative Balance Score

The OBS was calculated as the sum of seven pro-oxidant factors and six antioxidant factors selected based on previous studies [10,11,12,13,14,15,16,17]. The scheme of OBS is described in Table 1. Pro-oxidant factors include SFA, omega-6 PUFA, total iron intake, smoking status, drinking status, obesity status, and abdominal obesity status. Each question was scored 0, 1, or 2. The scores for SFA, omega-6 PUFA, and total iron intake were assigned 0 through 2 points according to the sex-specific tertile values of each variable corresponding to low (score 2), intermediate (score 1), and high (score 0). For smoking status, the scores for never smoker, former smoker, and current smoker were 2, 1, and 0, respectively. For drinking status, the scores for a non-drinker, mild drinker (1–19 g/day in men, 1–9 g/day in women), and moderate drinker (20–29 g/day in men, 10–19 g/day in women) were 2, 1, and 0, respectively. Zero points were given for people with obesity, one point was given for people who were overweight, and 2 points were given for people within a normal weight range. Zero points were given for people with abdominal obesity. Antioxidant factors include intakes of omega-3 PUFA, vitamin C, vitamin E, selenium, and beta-carotene and physical activity. The scores for omega-3 PUFA, vitamin C, vitamin E, selenium, and beta-carotene intake were assigned 0 through 2 points according to the sex-specific tertile values of each variable corresponding to high (score 2), intermediate (score 1), and low (score 0). Two points were given for high-intensity physical activity, one for moderate physical activity, and 0 for low physical activity. The sums of the OBSs ranged from 0 to 26 points. We classified the participants into sex-specific tertile groups according to OBSs.

### 2.3. Assessment of T2DM

T2DM was characterized as the presence of one or more of the following criteria: (1) a fasting plasma glucose level of 126 mg/dL or higher, (2) a 2 h after 75 g oral glucose tolerance test plasma glucose level of 200 mg/dL or higher, (3) glycosylated hemoglobin of 6.5% or higher, (4) treatment with oral anti-diabetic medicine, or (5) treatment with insulin therapy [20].

### 2.4. Covariates

A well-trained medical staff conducted health examinations and interviews according to a standard protocol. The detailed protocol of KoGES was available on the website (http://www.cdc.go.kr/contents.es?mid=a40504010000, accessed on 23 January 2023). Body mass index (BMI) was calculated as a person’s weight in kilograms divided by the square of height in meters. Overweight was defined as when a person’s BMI was 23 kg/m^2^ or higher, and obesity was defined as when a person’s BMI was 25 kg/m^2^ or higher, respectively, based on the 2018 Korean Society for the Study of Obesity (KSSO) guideline [21]. Abdominal obesity was defined as a person’s waist circumference (WC) being 90 cm or higher in men and 85 cm or higher in women, based on the 2018 KSSO guideline [21]. Mean blood pressure (MBP, mmHg) was calculated as diastolic blood pressure (DBP) + 1/3 × [systolic blood pressures (SBP)-DBP]. Information about smoking, alcohol consumption, physical activity, education level, and household income was obtained from the self-reported questionnaires. A participant who had never smoked or smoked less than 100 cigarettes in their lifetime was defined as a never smoker. A participant who quit smoking and smoked more than 100 cigarettes during their lifetime was defined as a former smoker. A participant who smoked currently and had smoked more than 100 cigarettes during their lifetime was defined as a current smoker. We calculated each participant’s daily alcohol intake (g/day). A heavy drinker was defined as a person who drinks alcohol more than 30 g/day in men and more than 20 g/day in women. A mild-to-moderate drinker was defined as a person who drinks alcohol below 30 g/day in men and below 20 g/day in women. A non-drinker was defined as a person who did not drink alcohol. Physical activity was measured as metabolic equivalent of task (MET)-hours per day (MET-h/day) using the International Physical Activity Questionnaire [22]. A nutrition survey was conducted through a face-to-face interview in an individual’s home. Total energy intake and nutritional status were calculated using a validated 103-item food frequency questionnaire [23]. We used the daily total energy intake (kcal/day), omega-6 PUFA, total iron (mg/day), SFA (g/day), omega-3 PUFA (g/day), selenium (µg/day), vitamin C (mg/day), vitamin E (mg/day), and beta-carotene (µg/day) intake. The educational levels were classified as elementary/middle school, high school, and college/university. Monthly household income was categorized into less than 100 million South Korean Won, 100–200 million South Korean Won, and more than 200 million South Korean Won. The plasma glucose, serum insulin, total cholesterol, triglyceride, high-density lipoprotein (HDL) cholesterol, and C-reactive protein (CRP) were measured after at least 8 h of fasting using a Hitachi 700-110 Chemistry Analyzer (Hitachi, Ltd., Tokyo, Japan).

### 2.5. Statistical Analysis

After the normality test, variables with normal distribution were presented as mean ± standard deviations, and those with non-normal distribution were represented as median (25th, 75th). Continuous variables were compared using the one-way analysis of variance or using the Kruskal–Wallis test according to the sex-specific OBS tertiles. All statistical analyses were performed in a sex-specific manner. Categorical variables were represented as a number (%) and compared using the chi-square test. To determine cumulative incidence T2DM according to the sex-specific OBS tertiles, Kaplan–Meier curves with the log-rank test were utilized. We calculated the hazard ratio (HR) and 95% confidence interval (CI) for incident T2DM in the sex-specific middle tertile (T2) and highest tertile (T3) groups compared with the referent lowest tertile (T1) group using univariable and multivariable Cox proportional hazard regression analyses. We included age, total energy intake, MBP, education level, household income, fasting plasma glucose, serum insulin, serum total cholesterol, serum triglyceride, and serum CRP levels in the adjusted model. All statistical analyses were performed with SAS software (version 9.4; SAS Institute Inc., Cary, NC, USA) and R software (version 4.1.1; R Foundation for Statistical Computing, Vienna, Austria). A *p*-value less than 0.05 was regarded as statistically significant.

## 3. Results

### 3.1. Baseline Characteristics of the Study Population

Table 2 shows the baseline characteristics of the study population according to the OBS tertiles in men and women. In men, the T1 group had higher levels of MBP (*p* = 0.001), serum glucose (*p* = 0.008), insulin (*p* < 0.001), total cholesterol (*p* < 0.001), triglyceride (*p* < 0.001), CRP (*p* < 0.001), and total energy intake (*p* < 0.001) and had lower levels of HDL cholesterol (*p* < 0.001). In women, the T1 group had higher levels of MBP (*p* < 0.001), serum glucose (*p* < 0.001), insulin (*p* < 0.001), total cholesterol (*p* < 0.001), triglyceride (*p* < 0.001), CRP (*p* < 0.001), and total energy intake (*p* < 0.001) and had lower levels of HDL cholesterol (*p* < 0.001). The proportion of higher education level and household income was significantly higher in the T3 group in both men and women.

Table 3 shows the baseline characteristics of individual components in relation to sex-specific OBS tertile groups. In both men and women, the T3 group had a higher SFA (*p* < 0.001), omega-6 PUFA (*p* < 0.001), total iron (*p* < 0.001), omega-3 PUFA (*p* < 0.001), vitamin C (*p* < 0.001), vitamin E (*p* < 0.001), selenium (*p* < 0.001), and beta-carotene intake (*p* < 0.001). In both men and women, those in the T1 group were more likely to be people with obesity (*p* < 0.001), current drinkers (*p* < 0.001), and current smokers (*p* < 0.001); had abdominal obesity (*p* < 0.001); and had lower physical activity (*p* < 0.001) than other groups.

### 3.2. Longitudinal Association of OBS and Incident T2DM

Throughout the average 13.6-year follow-up period, 908 (26.05%) men and 880 (22.66%) women developed new-onset T2DM.

**Figure 2:**
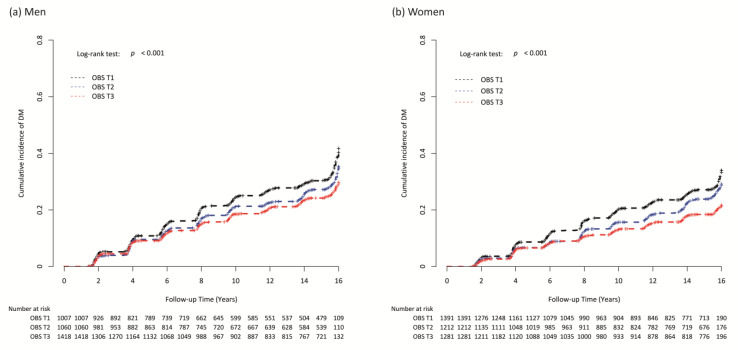
Kaplan–Meier curves for cumulative incidence of type 2 diabetes mellitus according to the sex-specific oxidative balance score tertiles in (**a**) men and (**b**) women.

Figure 2 presents the cumulative new-onset T2DM according to the sex-specific OBS tertiles as Kaplan–Meier curves. The T3 group showed the significantly lowest cumulative incident T2DM, followed by the T2 and T1 groups, in both men and women (both *p*-values for log-rank test < 0.001) (Figure 2a,b).

Table 4 shows the relationship between OBSs and incident T2DM in men and women. In men, the incidence rate per 1000 person-years was 27.49 in T1, 23.21 in T2, and 19.61 in T3. Compared with referent T1, the HR and 95% CI for new-onset T2DM were 0.85 (0.72–0.99) in T2 and 0.72 (0.62–0.85) in T3 (*p* for trend < 0.001). In the adjusted model, the HR and 95% CI for new-onset T2DM were 0.86 (0.73–1.02) in T2 and 0.83 (0.70–0.99) in T3 (p for trend = 0.035), compared with referent T1. In women, the incidence rate per 1000 person-years was 22.65 in T1, 19.09 in T2, and 14.48 in T3. The HR and 95% CI for new-onset T2DM were 0.84 (0.72–0.98) in T2 and 0.64 (0.54–0.75) in T3 (*p* for trend <0.001), compared with referent T1. The adjusted HR and 95% CI for new-onset T2DM were 0.94 (0.80–1.11) in T2 and 0.78 (0.65–0.94) in T3 (*p* for trend = 0.010), compared with referent T1. The HR and 95% CI for new-onset T2DM per one increment of OBS were 0.94 (0.91–0.96) in men and 0.91 (0.89–0.94) in women. Similar trends were shown in the adjusted model. 

## 4. Discussion

From this prospective study of a large, community-based Korean cohort over 16 years, OBSs were independently and inversely related to incident T2DM even after controlling confounding variables. 

In both men and women, the T3 group had 0.83- and 0.78-fold lower HRs for incident T2DM compared with T1 group, respectively. These findings agreed with the results of a previous cross-sectional study, which found that a higher OBS was positively related to better glycemic control in T2DM patients [24]. These data support the hypothesis that a healthy balance of pro- and antioxidant exposure has protection effect against T2DM. To the best of our knowledge, despite the associations between OBS and various health outcomes, including chronic kidney disease [25], hypertension [26], and metabolic syndrome [27], only one cross-sectional study found an association between OBS and glycemic control until the present [24]. A greater OBS, which denotes a predominance of antioxidant exposures over pro-oxidant exposures, has been associated with better glycemic control in Iranian people with T2DM, according to a prior study. [24]. In the prior study, the multivariable-adjusted mean HbA1c and FSG of participants in the highest tertile of OBS were noticeably lower than those in the lowest tertile (for HbA1c: mean difference—0.73 %; for FSG: mean difference—10.2 mg/dL; both *p* < 0.050). However, causal relationships cannot be inferred due to the study’s cross-sectional nature. This cross-sectional study was performed on participants who have already been diagnosed with T2DM. Our prospective study is the first approach to evaluate the effect of OBS on the incidence of T2DM in the general population.

In both men and women, the T3 group consumed higher amounts of both antioxidant components (such as omega-3 PUFA, selenium, vitamin C, vitamin E, and beta-carotene) and pro-oxidant components (such as saturated fatty acids, omega-6, and iron) compared with the other groups. This could potentially be attributed to their higher total energy intake. Considering these findings, it is believed that taking into account the OBS is more important than considering the individual components alone. Additionally, one important consideration is that factors like smoking, alcohol consumption, and obesity may have a greater impact on an OBS.

There are several persuasive mechanisms assisting the noted associations with lower risk for T2DM in the current study. Healthy diet patterns emphasizing a high consumption of fruits, vegetables, nuts, and fish are associated with health benefits including improvement of serum glucose and lipid level and weight loss [28]. Fruits, vegetables, nuts, and fish are rich sources of vitamins, minerals, polyphenols, and healthy fats, which have been associated with enhancing insulin sensitivity and reducing inflammation [29].

Physical activity yields a range of favorable effects, including enhancements in serum lipids, peripheral insulin sensitivity, reduction in blood pressure, mitigation of inflammation, and facilitation of weight loss [30]. Smoking can negatively impact pancreatic β-cell function and insulin sensitivity, promote inflammation, and contribute to increased visceral adiposity, in contrast to individuals who do not smoke [31]. Therefore, research groups have provided evidence that adopting a healthy lifestyle, encompassing reduced alcohol consumption, weight control, and increased vegetable intake, can effectively mitigate the risk of developing T2DM among individuals with impaired glucose tolerance and fasting glucose levels [32]. Further recent meta-analysis highlights that combining healthy lifestyles including healthy diet patterns, physical activity, cessation of smoking, and a healthy weight is closely associated with lower risk of T2DM [8].

This study has a few limitations. First, selection bias, as in other prospective studies, could have occurred. The subjects were recruited from 38 health examination centers and hospitals in the Republic of Korea’s urban district, and only those willing to perform were enrolled. We could not assess the effects of individual pro- and anti-inflammatory cytokines, including TNF-α, IL-1β, IL-4, IL-6, and IL-10. Second, there is no information in the KoGES on detailed prescriptions for antidiabetic medications. Third, in the KoGES dataset, only the baseline survey data for OBS values were utilized. This was because follow-up information specifically related to diet was unavailable. It is important to note that all variables included in the OBS have the potential to change over time. Therefore, future studies should consider analyzing the impact of changes in OBSs over time on the incidence of T2DM. Forth, each component comprising the oxidative balance score may exert unique effects on the incidence of T2DM. Therefore, it is crucial to employ an analytical approach that incorporates the weights associated with each pro-oxidant and antioxidant component when evaluating their influence on the development of T2DM. Further research is needed to clarify the association between OBS and T2DM. Finally, the indicators included in an OBS can contribute to the development of T2DM not only through oxidative stress effects but also through other mechanisms. For instance, high levels of physical activity have a protective effect against diabetes by improving insulin resistance in the muscles and liver [33]. On the other hand, obesity can contribute to T2DM through altered pancreatic hormone secretion, impaired glucose uptake in skeletal muscles, and hepatic insulin resistance [34]. Therefore, the group with high OBSs may have been influenced by additional mechanisms, beyond oxidative stress, in the occurrence of T2DM. Despite the above limitations, the most notable feature of this prospective study was confirmation of the incidence of T2DM by analyzing FFQ nutritional details on a large scale over 16 years. As a result, it reduces the possibility of recall bias and provides more reliable results than case-control studies. This current study is significant for providing evidence of a positive relationship between OBS and T2DM incidence risk. We anticipate that the present research will help lower the incidence of T2DM by highlighting the importance of an antioxidant-rich diet and drawing public attention to the risk of a pro-inflammatory lifestyle and diet.

## 5. Conclusions

We found that higher OBS was significantly related to a lower risk of T2DM among community-dwelling middle-aged and older Korean adults. Maintaining an optimal weight, physical activity, a non-smoking lifestyle, and a healthy diet pattern could be effective for lowering T2DM risk.

## Figures and Tables

**Table 1:** Oxidative balance score assignment scheme.

OBS Components	Assignment Scheme *
1. Saturated fatty acid [P]	0 = high (3rd tertile), 1 = intermediate (2nd tertile), 2 = low (1st tertile)
2. Omega-6 PUFA intake [P]	0 = high (3rd tertile), 1 = intermediate (2nd tertile), 2 = low (1st tertile)
3. Total iron intake [P]	0 = high (3rd tertile), 1 = intermediate (2nd tertile), 2 = low (1st tertile)
4. Smoking status [P]	2 = never smoker, 1 = former smoker, 0 = current smoker
5. Drinking status [P]	2 = non-drinker, 1 = mild-to-moderate drinker (<30 g/day in men, <20 g/day in women), 0 = heavy drinker (≥30 g/day in men, ≥20 g/day in women)
6. Overweight/obese [P]	2 = normal, 1 = overweight, 0 = obese
7. Abdominal obesity [P]	1 = normal, 0 = abdominal obesity
8. Omega-3 PUFA intake [A]	0 = low (1st tertile), 1 = intermediate (2nd tertile), 2 = high (3rd tertile)
9. Vitamin C intake [A]	0 = low (1st tertile), 1 = intermediate (2nd tertile), 2 = high (3rd tertile)
10. Vitamin E intake [A]	0 = low (1st tertile), 1 = intermediate (2nd tertile), 2 = high (3rd tertile)
11. Selenium intake [A]	0 = low (1st tertile), 1 = intermediate (2nd tertile), 2 = high (3rd tertile)
12. Total beta-carotene intake [A]	0 = low (1st tertile), 1 = intermediate (2nd tertile), 2 = high (3rd tertile)
13. Physical activity [A]	0 = low (<7.5 METs-h/wk), 1 = moderate (7.5–30 METs-h/wk), 2 = high (>30 METs-h/wk)

* Low, intermediate, and high categories correspond to sex-specific tertile values among participants in the KoGES at the baseline survey. Abbreviations: P, pro-oxidant; A, antioxidant; PUFA, poly-unsaturated fatty acid; MET, metabolic equivalent of task; KoGES, Korean Genome and Epidemiology Study.

**Table 2:** Baseline characteristics of the study population.

	Oxidative Balance Score							
	Men				Women			
Variables	T1 (*n* = 1007)	T2 (*n* = 1060)	T3 (*n* = 1418)	*p* *	T1 (*n* = 1391)	T2 (*n* = 1212)	T3 (*n* = 1281)	*p* *
Age, years	50.8 ± 8.5	51.8 ± 8.7	51.1 ± 8.7	0.501	53.9 ± 9.0	52.1 ± 8.9	50.0 ± 8.3	<0.001
MBP, mmHg	98.2 ± 12.0	98.1 ± 12.3	96.5 ± 12.7	0.001	97.4 ± 13.8	95.2 ± 13.6	91.7 ± 13.0	<0.001
Glucose, mg/dL	85.1 ± 9.4	84.9 ± 9.1	84.1 ± 8.7	0.008	81.9 ± 8.0	81.0 ± 7.6	80.5 ± 7.4	<0.001
Insulin, μU/mL	6.8 [4.8; 9.5]	6.5 [4.9; 8.9]	6.1 [4.7; 8.3]	<0.001	7.7 [5.8; 10.2]	7.3 [5.5; 9.9]	7.1 [5.3; 9.3]	<0.001
Total cholesterol, mg/dL	193.8 ± 34.5	191.7 ± 35.3	188.8 ± 34.1	<0.001	192.6 ± 34.0	189.7 ± 35.1	185.0 ± 32.0	<0.001
Triglyceride, mg/dL	168.0 [124.0; 231.5]	141.5 [109.0; 200.5]	129.0 [95.0; 182.0]	<0.001	134.0 [100.0; 182.0]	120.0 [91.5; 163.0]	111.0 [87.0; 150.0]	<0.001
HDL cholesterol, mg/dL	42.7 ± 9.6	43.7 ± 9.5	44.6 ± 10.5	<0.001	44.4 ± 9.7	46.4 ± 10.2	47.1 ± 9.9	<0.001
CRP, mg/dL	0.16 [0.08; 0.27]	0.15 [0.07; 0.26]	0.13 [0.06; 0.22]	<0.001	0.14 [0.08; 0.24]	0.14 [0.06; 0.23]	0.11 [0.04; 0.20]	<0.001
Education level, *n* (%)				0.595				<0.001
Elementary/middle school	427 (42.4%)	451 (42.7%)	569 (40.3%)		1052 (76.3%)	797 (66.3%)	724 (56.7%)	
High school	366 (36.4%)	378 (35.8%)	514 (36.4%)		274 (19.9%)	317 (26.4%)	436 (34.2%)	
College/university	213 (21.2%)	226 (21.4%)	330 (23.4%)		53 (3.8%)	88 (7.3%)	116 (9.1%)	
Household income, *n* (%)				0.005				<0.001
<100 million South Korean Won	259 (25.9%)	272 (25.8%)	380 (26.9%)		658 (48.1%)	474 (39.6%)	414 (32.9%)	
100–200 million South Korean Won	338 (33.8%)	286 (27.1%)	447 (31.7%)		375 (27.4%)	361 (30.2%)	345 (27.4%)	
>200 million South Korean Won	402 (40.2%)	496 (47.1%)	584 (41.4%)		336 (24.5%)	362 (30.2%)	498 (39.6%)	
Energy intake, kcal/day	1798.5 ± 487.5	1979.5 ± 650.1	2250.6 ± 748.2	<0.001	1607.3 ± 513.4	1876.1 ± 656.9	2211.7 ± 858.5	<0.001

* *p*-value for the comparison of the baseline characteristics among sex-specific tertile groups of oxidative balance scores at the baseline survey. Significance was set at *p* < 0.05. Abbreviations: MBP, mean blood pressure; CRP, C-reactive protein.

**Table 3:** Individual components of the score by oxidative balance score tertiles.

	Oxidative Balance Score							
	Men				Women			
Variables	T1 (*n* = 1007)	T2 (*n* = 1060)	T3 (*n* = 1418)	*p* *	T1 (*n* = 1391)	T2 (*n* = 1212)	T3 (*n* = 1281)	*p* *
Saturated fatty acid, g/day	8.7 ± 4.0	10.5 ± 6.2	12.9 ± 7.7	<0.001	7.9 ± 4.5	10.5 ± 6.3	13.9 ± 9.6	<0.001
Omega-6 PUFA intake, g/day	7.6 ± 4.0	8.6 ± 5.5	9.2 ± 5.4	<0.001	7.5 ± 4.1	8.6 ± 5.6	9.8 ± 6.4	<0.001
Total iron intake, mg/day	16.2 ± 6.1	19.3 ± 8.8	23.5 ± 10.9	<0.001	14.6 ± 6.2	18.8 ± 8.5	24.5 ± 12.9	<0.001
Smoking status, *n* (%)				<0.001				<0.001
Current smoker	647 (64.3%)	519 (49.0%)	471 (33.2%)		86 (6.2%)	30 (2.5%)	7 (0.5%)	
Former smoker	273 (27.1%)	352 (33.2%)	442 (31.2%)		27 (1.9%)	12 (1.0%)	4 (0.3%)	
Never smoker	87 (8.6%)	189 (17.8%)	505 (35.6%)		1278 (91.9%)	1170 (96.5%)	1270 (99.1%)	
Drinking status, *n* (%)				<0.001				<0.001
Heavy drinker	295 (29.3%)	200 (18.9%)	167 (11.8%)		37 (2.7%)	12 (1.0%)	8 (0.6%)	
Mild-to-moderate drinker	565 (56.1%)	588 (55.5%)	680 (48.0%)		433 (31.1%)	315 (26.0%)	236 (18.4%)	
Non-drinker	147 (14.6%)	272 (25.7%)	571 (40.3%)		921 (66.2%)	885 (73.0%)	1037 (81.0%)	
Obesity status, *n* (%)				<0.001				<0.001
Obese	596 (59.2%)	426 (40.2%)	322 (22.7%)		892 (64.1%)	523 (43.2%)	267 (20.8%)	
Overweight	228 (22.6%)	301 (28.4%)	411 (29.0%)		345 (24.8%)	298 (24.6%)	384 (30.0%)	
Normal weight	183 (18.2%)	333 (31.4%)	685 (48.3%)		154 (11.1%)	391 (32.3%)	630 (49.2%)	
Abdominal obesity, *n* (%)	350 (34.8%)	226 (21.3%)	126 (8.9%)	<0.001	755 (54.3%)	374 (30.9%)	199 (15.5%)	<0.001
Omega-3 PUFA intake, g/day	1.1 ± 0.6	1.3 ± 0.9	1.5 ± 0.9	<0.001	1.0 ± 0.6	1.3 ± 0.9	1.6 ± 1.1	<0.001
Vitamin C intake, mg/day	73.8 ± 45.1	109.4 ± 85.9	160.0 ± 116.9	<0.001	81.5 ± 64.8	135.1 ± 116.3	205.6 ± 149.7	<0.001
Vitamin E intake, mg/day	10.5 ± 4.0	13.6 ± 6.6	17.8 ± 8.5	<0.001	9.2 ± 4.4	13.6 ± 7.1	19.2 ± 10.5	<0.001
Selenium intake, μg/day	36.9 ± 18.5	49.5 ± 31.0	66.2 ± 38.4	<0.001	29.6 ± 18.6	46.0 ± 28.6	67.1 ± 47.8	<0.001
Beta-carotene intake, μg/day	2212.2 ± 1530.4	3409.7 ± 2930.3	4938.1 ± 3940.7	<0.001	1980.8 ± 1415.3	3283.4 ± 2557.0	5325.7 ± 4545.6	<0.001
Physical activity, *n* (%)				<0.001				<0.001
Low (<7.5 METs-h/day)	103 (10.2%)	51 (4.8%)	47 (3.3%)		197 (14.2%)	97 (8.0%)	63 (4.9%)	
Moderate (7.5–30 METs-h/day)	682 (67.7%)	629 (59.3%)	774 (54.6%)		847 (60.9%)	787 (64.9%)	770 (60.1%)	
High (>30 METs-h/day)	222 (22.0%)	380 (35.8%)	597 (42.1%)		347 (24.9%)	328 (27.1%)	448 (35.0%)	

* *p*-value for the comparison of the baseline characteristics among sex-specific tertile groups of oxidative balance score at the baseline survey. Significance was set at *p* < 0.05. Abbreviations: PUFA, poly-unsaturated fatty acid; MET, metabolic equivalent of task.

**Table 4:** Cox proportional hazard regression analysis presenting the relationship of oxidative balance scores with incident type 2 diabetes mellitus.

Oxidative Balance Score Tertiles	Numbers, *n*	New-Onset Cases, *n*	Follow-Up Period, Person-Year	Incidence Rate Per 1000 Person-Years	Unadjusted	Adjusted
					HR (95% CI)	HR (95% CI)
Men						
Continuous (per 1 increment)					0.94 (0.91–0.96)	0.96 (0.94–0.99)
T1	1007	306	11,130.3	27.49	1 (reference)	1 (reference)
T2	1060	284	12,238.5	23.21	0.85 (0.72–0.99)	0.86 (0.73–1.02)
T3	1418	318	16,218.5	19.61	0.72 (0.62–0.85)	0.83 (0.70–0.99)
*p* for trend					<0.001	0.035
Women						
Continuous (per 1 increment)					0.91 (0.89–0.94)	0.95 (0.92–0.98)
T1	1391	367	16,206.6	22.65	1 (reference)	1 (reference)
T2	1212	281	14,717.9	19.09	0.84 (0.72–0.98)	0.94 (0.80–1.11)
T3	1281	232	16,025.8	14.48	0.64 (0.54–0.75)	0.78 (0.65–0.94)
*p* for trend					<0.001	0.010

Adjusted for age, total energy intake, mean blood pressure, education level, household income, plasma fasting glucose, serum insulin, serum total cholesterol, serum triglyceride, and serum C-reactive protein level. Abbreviations: HR, hazard ratio; CI, confidence interval.

## Data Availability

The Korean Genome and Epidemiology Study data are available through a procedure described at https://nih.go.kr/ko/main/main.do (accessed on 23 January 2023).

## References

[1] Saeedi P., Petersohn I., Salpea P., Malanda B., Karuranga S., Unwin N., Colagiuri S., Guariguata L., Motala A.A., Ogurtsova K. (2019). Global and regional diabetes prevalence estimates for 2019 and projections for 2030 and 2045: Results from the international diabetes federation diabetes atlas, 9(th) edition. Diabetes Res. Clin. Prac..

[2] Bae J.C. (2018). Trends of diabetes epidemic in korea. Diabetes Metab. J..

[3] Oh S.-H., Ku H., Park K.S. (2021). Prevalence and socioeconomic burden of diabetes mellitus in south korean adults: A population-based study using administrative data. BMC Public Health.

[4] Cousin E., Schmidt M.I., Ong K.L., Lozano R., Afshin A., Abushouk A.I., Agarwal G., Agudelo-Botero M., Al-Aly Z., Alcalde-Rabanal J.E. (2022). Burden of diabetes and hyperglycaemia in adults in the americas, 1990–2019: A systematic analysis for the global burden of disease study 2019. Lancet Diabetes Endocrinol..

[5] Galicia-Garcia U., Benito-Vicente A., Jebari S., Larrea-Sebal A., Siddiqi H., Uribe K.B., Ostolaza H., Martin C. (2020). Pathophysiology of type 2 diabetes mellitus. Int. J. Mol. Sci..

[6] Wu H., Ballantyne C.M. (2020). Metabolic inflammation and insulin resistance in obesity. Circ. Res..

[7] Rohm T.V., Meier D.T., Olefsky J.M., Donath M.Y. (2022). Inflammation in obesity, diabetes, and related disorders. Immunity.

[8] Khan T.A., Field D., Chen V., Ahmad S., Mejia S.B., Kahleová H., Rahelić D., Salas-Salvadó J., Leiter L.A., Uusitupa M. (2023). Combination of multiple low-risk lifestyle behaviors and incident type 2 diabetes: A systematic review and dose-response meta-analysis of prospective cohort studies. Diabetes Care.

[9] Goodman M., Bostick R.M., Dash C., Flanders W.D., Mandel J.S. (2007). Hypothesis: Oxidative stress score as a combined measure of pro-oxidant and antioxidant exposures. Ann. Epidemiol..

[10] Hernández-Ruiz Á., García-Villanova B., Guerra-Hernández E., Amiano P., Ruiz-Canela M., Molina-Montes E. (2019). A review of a priori defined oxidative balance scores relative to their components and impact on health outcomes. Nutrients.

[11] Lakkur S., Goodman M., Bostick R.M., Citronberg J., McClellan W., Flanders W.D., Judd S., Stevens V.L. (2014). Oxidative balance score and risk for incident prostate cancer in a prospective u.S. Cohort study. Ann. Epidemiol..

[12] Kong S.Y., Goodman M., Judd S., Bostick R.M., Flanders W.D., McClellan W. (2015). Oxidative balance score as predictor of all-cause, cancer, and noncancer mortality in a biracial us cohort. Ann. Epidemiol..

[13] Cho A.R., Kwon Y.J., Lim H.J., Lee H.S., Kim S., Shim J.Y., Lee H.R., Lee Y.J. (2018). Oxidative balance score and serum γ-glutamyltransferase level among korean adults: A nationwide population-based study. Eur. J. Nutr..

[14] Haggag Mel S., Elsanhoty R.M., Ramadan M.F. (2014). Impact of dietary oils and fats on lipid peroxidation in liver and blood of albino rats. Asian Pac. J. Trop Biomed.

[15] Pitaraki E.E. (2017). The role of mediterranean diet and its components on the progress of osteoarthritis. J. Frailty Sarcopenia Falls.

[16] Romeu M., Aranda N., Giralt M., Ribot B., Nogues M.R., Arija V. (2013). Diet, iron biomarkers and oxidative stress in a representative sample of mediterranean population. Nutr. J..

[17] Valenzuela R., Rincón-Cervera M., Echeverría F., Barrera C., Espinosa A., Hernández-Rodas M.C., Ortiz M., Valenzuela A., Videla L.A. (2018). Iron-induced pro-oxidant and pro-lipogenic responses in relation to impaired synthesis and accretion of long-chain polyunsaturated fatty acids in rat hepatic and extrahepatic tissues. Nutrition.

[18] Annor F.B., Goodman M., Okosun I.S., Wilmot D.W., Il’yasova D., Ndirangu M., Lakkur S. (2015). Oxidative stress, oxidative balance score, and hypertension among a racially diverse population. J. Am. Soc. Hypertens.

[19] Kim Y., Han B.G. (2017). Cohort profile: The korean genome and epidemiology study (koges) consortium. Int. J. Epidemiol..

[20] American Diabetes Association (2020). 2. Classification and diagnosis of diabetes: Standards of medical care in diabetes-2020. Diabetes Care.

[21] Seo M.H., Lee W.Y., Kim S.S., Kang J.H., Kang J.H., Kim K.K., Kim B.Y., Kim Y.H., Kim W.J., Kim E.M. (2019). 2018 korean society for the study of obesity guideline for the management of obesity in korea. J. Obes. Metab. Syndr..

[22] Oh J.Y., Yang Y.J., Kim B.S., Kang J.H. (2007). Validity and reliability of korean version of international physical activity questionnaire (ipaq) short form. J. Korean Acad. Fam. Med..

[23] Ahn Y., Kwon E., Shim J.E., Park M.K., Joo Y., Kimm K., Park C., Kim D.H. (2007). Validation and reproducibility of food frequency questionnaire for korean genome epidemiologic study. Eur. J. Clin. Nutr..

[24] Golmohammadi M., Ayremlou P., Zarrin R. (2021). Higher oxidative balance score is associated with better glycemic control among iranian adults with type-2 diabetes. Int. J. Vitam. Nutr. Res..

[25] Son D.H., Lee H.S., Seol S.Y., Lee Y.J., Lee J.H. (2023). Association between the oxidative balance score and incident chronic kidney disease in adults. Antioxidants.

[26] Lee J.H., Son D.H., Kwon Y.J. (2022). Association between oxidative balance score and new-onset hypertension in adults: A community-based prospective cohort study. Front. Nutr..

[27] Noruzi Z., Jayedi A., Farazi M., Asgari E., Dehghani Firouzabadi F., Akbarzadeh Z., Djafarian K., Shab-Bidar S. (2021). Association of oxidative balance score with the metabolic syndrome in a sample of iranian adults. Oxid. Med. Cell Longev..

[28] Chiavaroli L., Viguiliouk E., Nishi S.K., Blanco Mejia S., Rahelić D., Kahleová H., Salas-Salvadó J., Kendall C.W., Sievenpiper J.L. (2019). Dash dietary pattern and cardiometabolic outcomes: An umbrella review of systematic reviews and meta-analyses. Nutrients.

[29] Thomas M.S., Calle M., Fernandez M.L. (2023). Healthy plant-based diets improve dyslipidemias, insulin resistance, and inflammation in metabolic syndrome. A narrative review. Adv. Nutr..

[30] Chow L.S., Gerszten R.E., Taylor J.M., Pedersen B.K., van Praag H., Trappe S., Febbraio M.A., Galis Z.S., Gao Y., Haus J.M. (2022). Exerkines in health, resilience and disease. Nat. Rev. Endocrinol..

[31] Yeh H.C., Duncan B.B., Schmidt M.I., Wang N.Y., Brancati F.L. (2010). Smoking, smoking cessation, and risk for type 2 diabetes mellitus: A cohort study. Ann. Intern. Med..

[32] Davies M.J., Aroda V.R., Collins B.S., Gabbay R.A., Green J., Maruthur N.M., Rosas S.E., Del Prato S., Mathieu C., Mingrone G. (2022). Management of hyperglycemia in type 2 diabetes, 2022. A consensus report by the american diabetes association (ada) and the european association for the study of diabetes (easd). Diabetes Care.

[33] Kirwan J.P., Sacks J., Nieuwoudt S. (2017). The essential role of exercise in the management of type 2 diabetes. Cleve Clin. J. Med..

[34] Kahn S.E., Hull R.L., Utzschneider K.M. (2006). Mechanisms linking obesity to insulin resistance and type 2 diabetes. Nature.

